# IL-10 Is Critically Involved in Mycobacterial HSP70 Induced Suppression of Proteoglycan-Induced Arthritis

**DOI:** 10.1371/journal.pone.0004186

**Published:** 2009-01-14

**Authors:** Lotte Wieten, Suzanne E. Berlo, Corlinda B. ten Brink, Peter J. van Kooten, Mahavir Singh, Ruurd van der Zee, Tibor T. Glant, Femke Broere, Willem van Eden

**Affiliations:** 1 Institute of Infectious Diseases and Immunology, Division of Immunology, Utrecht University, Utrecht, the Netherlands; 2 LIONEX Diagnostics & Therapeutics GmbH, Braunschweig, Germany; 3 Section of Molecular Medicine, Department of Orthopedic Surgery, Rush University Medical Center, Chicago, Illinois, United States of America; University of Sheffield, United Kingdom

## Abstract

**Background:**

The anti-inflammatory capacity of heat shock proteins (HSP) has been demonstrated in various animal models of inflammatory diseases and in patients. However, the mechanisms underlying this anti-inflammatory capacity are poorly understood. Therefore, the possible protective potential of HSP70 and its mechanisms were studied in proteoglycan (PG) induced arthritis (PGIA), a chronic and relapsing, T cell mediated murine model of arthritis.

**Methodology/Principal Findings:**

HSP70 immunization, 10 days prior to disease induction with PG, inhibited arthritis both clinically and histologically. In addition, it significantly reduced PG-specific IgG2a but not IgG1 antibody production. Furthermore, IFN-γ and IL-10 production upon *in vitro* restimulation with HSP70 was indicative of the induction of an HSP70-specific T cell response in HSP70 immunized mice. Remarkably, HSP70 treatment also modulated the PG-specific T cell response, as shown by the increased production of IL-10 and IFN-γ upon *in vitro* PG restimulation. Moreover, it increased IL-10 mRNA expression in CD4^+^CD25^+^ cells. HSP70 vaccination did not suppress arthritis in IL-10^−/−^ mice, indicating the crucial role of IL-10 in the protective effect.

**Conclusions/Significance:**

In conclusion, a single mycobacterial HSP70 immunization can suppress inflammation and tissue damage in PGIA and results in an enhanced regulatory response as shown by the antigen-specific IL-10 production. Moreover, HSP70 induced protection is critically IL-10 dependent.

## Introduction

Heat shock proteins (HSP) are highly immunogenic and have the potential to trigger immuno-regulatory pathways. Although the exact mechanisms of immuno-regulation by HSP remain to be clarified, T cells specific for HSP were suggested to be involved in regulation of multiple chronic inflammatory diseases like rheumatoid arthritis (RA), diabetes and atherosclerosis [1]–[4].

Recent studies on HSP-responding T cells of juvenile idiopathic patients implied a regulatory role and contribution to disease remission for HSP60-specific T cells [5], [6]. Among the various HSP families of molecules, the immuno-modulatory potential of HSP60 has been studied most extensively. However, also other HSP have immuno-regulatory functions. For example treatment with bacterial HSP70 has been shown to protect against development of adjuvant induced arthritis in rats, whereas treatment with other highly conserved bacterial proteins did not [7]. Exposure to bacterial HSP has been shown to activate self HSP-specific T cells that were cross reactive with bacterial HSP and induced suppression of arthritis [8], [9]. In addition, a T cell line specific for an HSP70-derived peptide has been described to decrease disease severity upon transfer [10]. The protective potential of HSP, in animal models, has been reproduced by recent clinical trials with HSP-derived peptides in patients [11], [12].

Even though HSP induced immuno-regulation has been studied rather extensively, the mechanism of HSP induced protection in autoimmune diseases is still not clear. A potential regulatory mechanism might be via the induction of IL-10 since the important role of IL-10 in dampening inflammation has been described extensively [13]–[15]. Additionally, IL-10 requirement for their suppressive function has been thought to be a common feature among most subsets of regulatory T cells as summarized [16].

In the present study the protective potential of mycobacterial HSP70 immunization on inflammatory disease and its dependency on IL-10 were assessed in the proteoglycan-induced arthritis model (PGIA), a progressive T cell dependent, antibody-mediated murine model for RA [17]. In this model arthritis can be induced by immunization with human proteoglycan (PG) mixed with synthetic adjuvant dimethyldioctadecylammonium bromide (DDA) instead of complete Freund's adjuvant (CFA) [18]. Therefore, the possibility of interfering with immune responses induced by mycobacterial HSP present in CFA can be excluded. We found that HSP70 immunization dramatically suppressed arthritis development, subsequent tissue damage and the pathogenic PG-specific antibody response. Moreover, we demonstrate that HSP70 immunization results in a regulatory T cell response not only to HSP70, but also to the disease inducing PG. Finally, HSP70 did not suppress arthritis in IL-10 deficient mice, indicating that the regulatory response is IL-10 dependent.

## Materials and Methods

### Mice

Female BALB/c mice, retired breeders aged between 16–26 weeks, purchased from Charles River (Maastricht, The Netherlands), and IL-10^−/−^ mice also on the BALB/c background (kindly provided by Dr. D. Reddick, DNAX, Palo Alto, CA) [19] were bred, housed and fed under standard conditions. Experiments were approved by the Animal Experiment Committee of Utrecht University (Utrecht, the Netherlands).

### Induction and assessment of arthritis

Arthritis was induced with PG using a standard immunization protocol as described [17], [18]. Briefly, 300 µg PG protein was given by intra peritoneal injection (i.p) with 2 mg of DDA (Sigma, Zwijndrecht, the Netherlands) emulsified in 200 µl PBS on days 0 and 21. PG was prepared as described elsewhere [20]. After the second PG immunization the paws of mice were examined in a blinded fashion 3 times a week to record arthritic changes of the joints. The onset and severity of arthritis were determined using a visual scoring system based on swelling and redness of paws as described [18]. Upon sacrifice, joints were fixed in 10% buffered formalin, decalcified in 0.33 M neutralized EDTA, embedded in paraffin and 5 µm sagittal sections were stained with hematoxylin and eosin.

### HSP70 or control immunization

HSP70 or control immunization was done, 10 days prior to arthritis induction, by intraperitoneal (i.p.) immunization with 100 µg recombinant HSP70 of *Mycobacterium tuberculosis* (Mt) (LIONEX Diagnostics&Therapeutics GmbH, Braunschweig, Germany), or 100 µg control protein, either recombinant enhanced green fluorescent protein (EGFP) or ovalbumin (Ova) (Sigma) in adjuvant DDA, 2 mg emulsified in PBS or with PBS in a total volume of 200 µl. To avoid interference of LPS contamination with HSP70 treatment, HSP70 containing less than 2.1 EU/mg protein was used.

### Analysis of PG- and HSP70-specific serum antibody production

Antigen-specific serum antibody levels were determined by ELISA as described previously [18]. In brief, 96 well plates (Corning B.V. Live Sciences, Schiphol Rijk, the Netherlands) were coated by overnight incubation with 100 µl PG or HSP70 at 5 µg/ml in coating buffer (0.1 M NaHCO_3_, Na_2_CO_3_ pH 9.6). Free binding sites were blocked with blocking buffer, Roche blocking reagents for ELISA (Roche Diagnostics, Alkmaar, the Netherlands) followed by incubation with sera at increasing dilutions and subsequently peroxidase-conjugated anti-IgG1, -IgG2a or -total IgG (BD Pharmingen, Breda, the Netherlands) in blocking buffer. After ABTS incubation serum PG-specific antibody levels were calculated as OD relative to the OD measured for the corresponding isotypes of a standard of pooled sera from arthritic mice. For HSP70-specific antibody production a reference serum of HSP70 immunized mice was used. Data were expressed relative to the average of the control group.

### Measurement of antigen-specific T cell responses

Single-cell suspensions of spleens were cultured in triplicates in 96-well flat bottom plates (Corning B.V.) at 2×10^5^ cells per well, in the presence or absence of HSP70 (10 µg/ml), PG (10 µg/ml) or Ova (10 µg/ml). Iscove's Modified Dulbecco's Medium (IMDM) supplemented with 10% FCS (Bodinco B.V., Alkmaar, the Netherlands), 2 mM L-glutamine, 100 units/ml penicillin, 100 µg/ml streptomycin, and 5×10^−5^ M 2-mercaptoethanol was used as culture medium. After 72 or 96 hours, the cells were pulsed overnight with ^3^H-thymidine (0.4 µCi per well; Amersham Biosciences Europe GmbH, Roosendaal, the Netherlands), harvested and ^3^H uptake was measured by liquid scintillation counting (Microbeta, Perkin-Elmer Inc., Boston, MA). Supernatants of antigen stimulated spleen cell cultures were collected for cytokine assays after 72 hours. Supernatants harvested from cultures with cells isolated from WT mice were analyzed for IL-10 and interferon-gamma (IFN-γ) expression simultaneously by multiplex analysis using the Luminex 100 system (Becton Dickinson, Mountain View, CA). The LINCOplex assay was performed according to the manufacturer's instructions (Linco Research, Inc., St. Charles, Missouri). The concentrations of IL-10 and IFN-γ in the supernatants were calculated using LMAT software (Luminex Corporation, Austin, TX). IFN-γ secretion by cells isolated from IL10^−/−^ mice was measured by ELISA (BD OptEIA) according to manufactures protocol (BD Pharmingen).

### Assessment of IL-10 and Foxp3 mRNA expression, in CD4^+^CD25^+^ spleen cells

Isolation of CD4^+^CD25^+^ spleen cells from arthritic mice was done by staining single cell suspensions of spleen cells with anti-CD4-allophycocyanin (RM4-5) and anti-CD25 r-phycoerythrin (PC61) monoclonal antibodies (BD Pharmingen). CD4^+^CD25^+^ cells were sorted by CD4 and CD25 expression with a FACSVantage SE (Becton Dickinson). Then total mRNA extraction, with the RNeasy kit (Qiagen Benelux B.V., Venlo, the Netherlands), on column DNAse treatment (Qiagen), and transcription into cDNA using the iScript™ cDNA Synthesis Kit (Bio-Rad Laboratries B.V., Veenendaal, The Netherlands) were carried out according to manufacturers protocol. PCR (3 min at 95°C and 40 cycles of 10 s 95°C and 45 s at 59.5°C) and Real-Time detection were performed in a Bio-Rad MyiQ iCycler (Bio-Rad). Amplification was done using IQ™ SYBR Green® Supermix (Bio-Rad) with 0.25 µM final concentrations of primers specific for IL-10 (5′-GGT TGC CAA GCC TTA TCG GA-3′ and 5′-ACC TGC TCC ACT GCC TTG CT-3′), Foxp3 (5′- CCC AGG AAA GAC AGC AAC CTT and 5′-TTC TCA CAA CCA GGC CAC TTG-3) and hypoxanthine-guanine phosphoribosyl transferase (HPRT) (5′-CTG GTG AAA AGG ACC TCT CG and 5′-TGA AGT ACT CAT TAT AGT CAA GGG CA-3′). For each sample mRNA expression was normalized to the detected Ct value of HPRT and expressed relative to the average of the control group.

### Statistical analysis

Unless stated otherwise, data are expressed as mean±standard error of the mean (SEM). Statistical analysis was carried out using the Mann-Whitney U test (two-tailed) using Prism software (version 3.00, Graphpad Software Inc., San Diego). Significance level was set at (p<0.05).

## Results

### Immunization with HSP70 decreases arthritis incidence and severity

**Figure 1:**
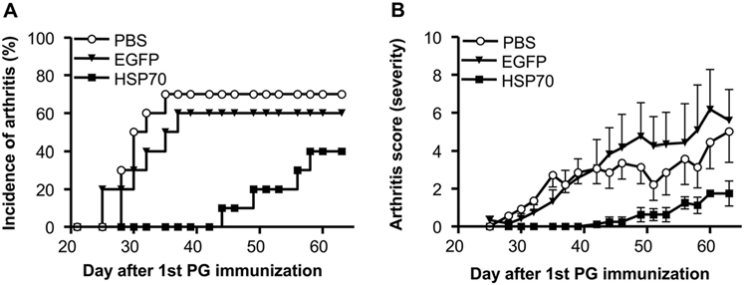
Decreased arthritis incidence and severity after HSP70 immunization. Mice were injected intraperitoneally, 10 days prior to disease induction, with either 100 µg recombinant microbial heat shock protein 70 (HSP70) or 100 µg enhanced green fluorescent protein (EGFP) both emulsified in synthetic adjuvant DDA or with PBS. Arthritis was induced by two immunizations of proteoglycan (PG) in DDA on day 0 and 21. (A) Arthritis incidence is expressed as the cumulative percentage of arthritic animals and (B) arthritis severity is expressed as the mean arthritis score of sick mice±SEM. Data are representative of three independent experiments (n = 10 in each group).

**Figure 2:**
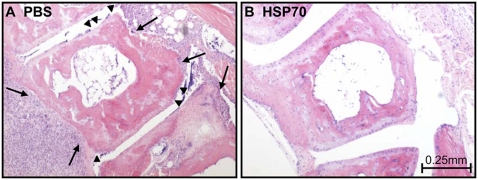
Decreased leukocyte infiltration and cartilage breakdown in HSP70 immunized mice. PBS or heat shock protein 70 (HSP70)/DDA immunization, followed by arthritis induction, was performed as described in figure 1. Upon sacrifice on day 35, histology of tarso-metatarsal joints was done by staining of the sections with hematoxylin and eosin. (A) Synovial hyperplasia with infiltrating leukocytes, cartilage- (arrow heads) and bone-erosions (arrows) can be seen. (B) In contrast, only a very few leukocytes, and no synovial cell proliferation or cartilage destruction were observed in the joint sections of HSP70 immunized mice.

To investigate the immuno-regulatory mechanisms of HSP70 in inflammatory diseases, the effect of mycobacterial HSP70 immunization in PGIA was studied. Mice were treated with HSP70 10 days prior to induction of arthritis by i.p. immunization with 100 µg HSP70, in the synthetic adjuvant DDA, whereas control groups received 100 µg EGFP or PBS in DDA. Subsequently, arthritis was induced by two i.p. immunizations with PG in DDA on day 0 and 21. HSP70 immunization lowered the incidence of arthritis and significantly delayed the onset of arthritis; day 52±3.2 in HSP70 immunized mice compared to day 31±2.0 in EGFP and 30±1.0 in PBS pretreated mice (p<0.01) (Fig. 1A). Furthermore, HSP70 pre-treatment evidently reduced severity of PGIA (Fig. 1B). In association, histological analysis of joint sections of HSP70 immunized arthritic mice showed very mild leukocyte infiltration, less reactive synovial cell proliferation, and consequently almost no cartilage damage compared to joint sections of control animals (Fig. 2A–B).

### Altered antigen-specific B cell responses after HSP70 immunization

**Figure 3:**
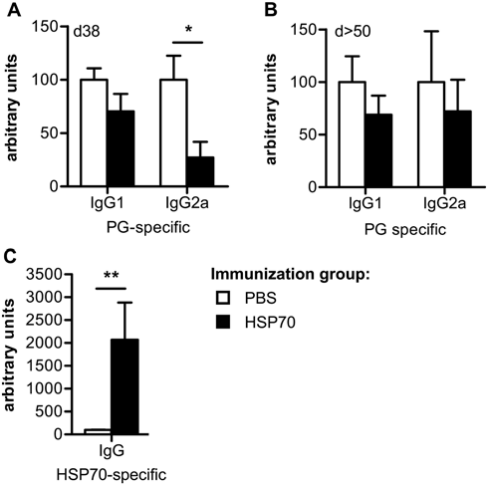
Altered PG- and HSP70-specific B cell responses in HSP70 immunized mice. Proteoglycan (PG)- or heat shock protein 70 (HSP70)-specific antibody production in the sera of mice was assessed by ELISA and relative serum antibody levels were calculated as OD relative to the OD measured for a standard of pooled sera. (A–B) PG-specific IgG1 and IgG2a levels were determined (A) on day 38 and (B) on day 50 or 64 after disease induction. (C) HSP70-specific total IgG production was measured in sera obtained on day 38. Values in A and C represent the mean±SEM (n = 5 mice in each group) and are representative for three independent experiments (PG-specific IgG) or one experiment (HSP70-specific IgG). Values in B represent mean±SEM (n = 6 mice) of sera obtained on either day 50 or day 64, no obvious difference between sera collected on day 50 or 64 was detected. Open bars show the PBS immunized group and filled bars the HSP70 immunized group. Significant difference (* p<0.05 or **p<0.01, Mann-Whitney U Test) is indicated by the asterisk.

PG-specific B cell responses have been described to be required for development of severe PGIA [17]. Therefore, the PG-specific B cell response and the effect of HSP70 immunization on this response were studied. On day 38 after the first PG injection PG-specific serum IgG1 and IgG2a levels were determined by ELISA. High IgG1 levels confirmed the induction of a B cell response against the disease inducing PG in both treatment groups. However, the level of IgG2a was lowered in HSP70 compared to PBS immunized mice (Fig. 3A). In addition, we studied the pattern on days 50–64, after development of mild disease in HSP70 immunized mice. At days 50–64 the difference between PBS and HSP70 immunized mice in IgG1 levels was comparable to day 38 but the decrease in IgG2a, as observed on day 38, was no longer significant (Fig. 3B). The data indicate that mainly the PG-specific IgG2a production was influenced by HSP70 immunization and underline the importance of this isotype for induction of PGIA. Recently, HSP-specific antibodies have been shown to play a role in HSP induced regulation in models for RA [21], [22]. To address whether HSP70-specific antibodies were also induced upon HSP70 immunization in the PGIA model, HSP70 serum antibody levels were analyzed by ELISA. Significantly increased HSP70-specific total IgG production was found in HSP70 immunized mice (Fig. 3C). Thus, both PG- and HSP70-specific B cell responses were altered upon HSP70 treatment as shown by a decreased pathogenic PG-specific response and an increased, possibly protective HSP70-specific B cell response.

### Increased antigen-specific proliferation, IL-10 and IFN-γ production after immunization with HSP70

**Figure 4:**
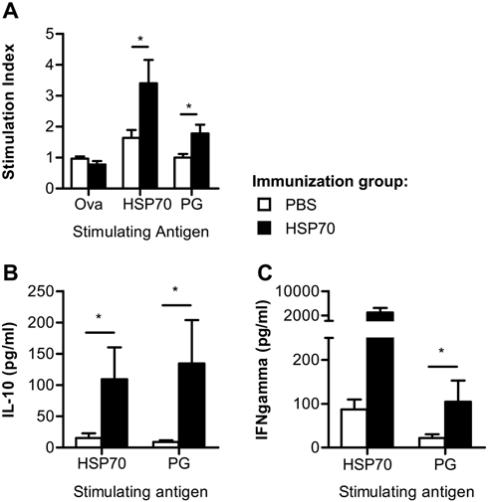
Augmented antigen-specific proliferation, IL-10 and IFN-γ production in HSP70 immunized mice. Mice were immunized with PBS or heat shock protein 70 (HSP70) and arthritis was induced as described in figure 1. Spleen cells were isolated on day 35 and cultured (2×10^5^/well) in the presence of recombinant microbial HSP70, proteoglycan (PG) or ovalbumin (Ova) (all at 10 µg/ml). (A) After 96 hours incubation proliferation was determined by measuring ^3^H incorporation. Results are expressed as mean stimulation index (CPM Antigen/CPM background)±SEM. The mean background values (responses without antigen) were for the PBS control group 8390±2135 and for the HSP70 group 10 180±2741. (B–C) For *in vitro* cytokine secretion the spleen cells were cultured in the presence of antigen. After 72 hours supernatants were harvested and assayed by multiplex analysis for IL-10 and IFN-γ excretion. Open bars show the PBS immunized group and filled bars the HSP70 immunized group. Values represent the mean±SEM of cytokine levels. Significant difference (p<0.05, Mann-Whitney U Test) is indicated by the asterisk. Data are representative for at least two independent experiments.

In previous studies, HSP-specific T cell responses have been described to be involved in HSP-induced protection from disease development [7], [9]. To study the effect of HSP70 immunization on differentiation of antigen-specific T cell responses, PG- and HSP70-specific proliferation and cytokine profiles were analyzed. Therefore, spleen cells from HSP70 or PBS immunized mice were harvested on day 35 after the first PG immunization. Antigen-specific proliferation was assessed by culturing the isolated cells for 96 hours with PG or HSP70 while Ova was used as control antigen. During the last 18 hours ^3^H-thymidine was added and incorporation was measured and expressed as mean stimulation index (CPM Antigen/CPM background)±SEM. The mean background values (responses without antigen) were for the PBS control group 8390±2135 and for the HSP70 group 10 180±2741. A significantly enhanced proliferative response to HSP70 restimulation was seen in HSP70 immunized mice as compared to control mice (Fig. 4A). Remarkably, also the proliferative response against the arthritis inducing PG was enhanced in the HSP70 group. Next, antigen-induced production of IL-10 and IFN-γ was studied in supernatants collected after 72 hours culturing in the presence of PG or HSP70. Increased IL-10 production, by spleen cells from HSP70 immunized mice, could be detected after HSP70 *in vitro* restimulation (Fig. 4B). Interestingly, this increased IL-10 production was also found after PG *in vitro* restimulation. Furthermore, the amount of IFN-γ produced by HSP70- and PG-specific cells from HSP70 treated mice was increased (Fig. 4C). In summary this clearly showed that HSP70 immunization not only induced an HSP70-specific T cell response but also modulated the T cell response to the disease inducing antigen.

### Enhanced IL-10 mRNA expression in CD4^+^CD25^+^ T cells after HSP70 immunization

**Figure 5:**
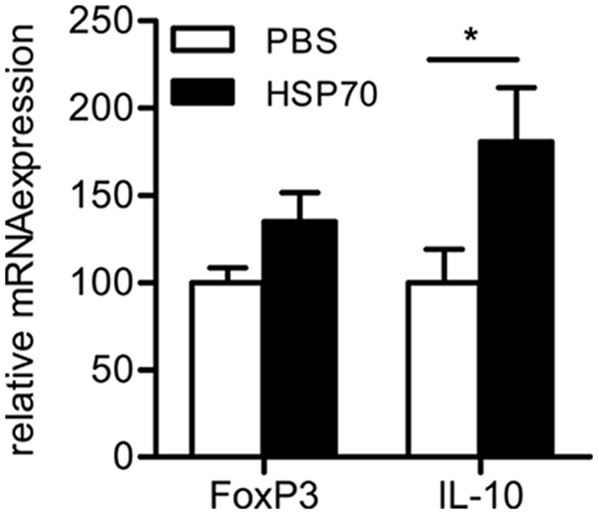
Increased IL-10 mRNA expression in CD4^+^CD25^+^ cells of HSP70 immunized mice. Mice were PBS or heat shock protein 70 (HSP70) immunized before induction of arthritis as described in figure 1. On day 38, CD4^+^CD25^+^ cells were isolated from the spleen followed by isolation of mRNA. Foxp3 and IL-10 mRNA expression was determined by Quantitative Real-Time PCR analysis. Data are normalized and expressed relative to HPRT and depicted as mean±SEM (n = 5 mice in each group). Significant difference (p<0.05, Mann-Whitney U Test) is indicated by the asterisk.

It has been proposed that induction or activation of HSP70-specifc regulatory T cells cross-reactive with self-HSP70 are involved in protection seen after HSP70 immunization [8], [9]. To study in more detail whether HSP70 immunization influenced natural regulatory T cells, CD4^+^CD25^+^ cells were isolated from the spleen on day 38 after the first PG injection. In this population Foxp3 and IL-10 mRNA expression were analyzed by Quantitative Real-Time PCR analysis showing that IL-10 mRNA expression was increased in the CD4^+^CD25^+^ population from HSP70 treated mice compared to PBS treated control mice (Fig. 5). No clear difference in Foxp3 mRNA expression was observed, between control and HSP70 immunized mice. This could be confirmed by intracellular staining and flow cytometric analysis of Foxp3 protein expression (data not shown). Indicating that HSP70 does not increase the population of Foxp3 expressing regulatory T cells.

### IL-10 is important for the protective effects of HSP70 immunization

**Figure 6:**
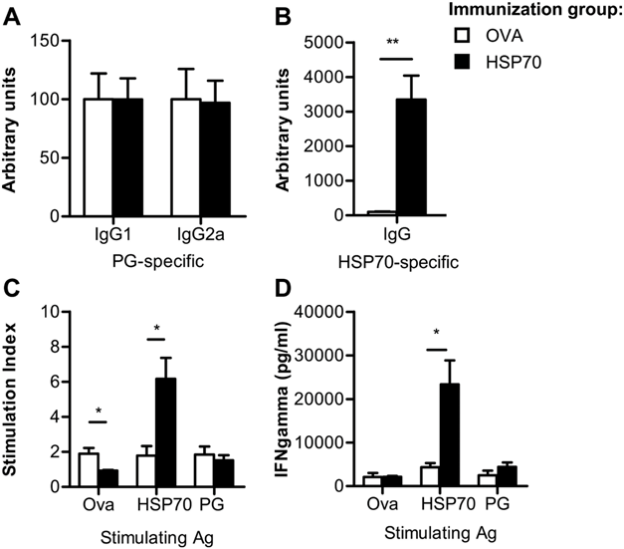
Protective effects found upon HSP70 immunization are IL-10 dependent. IL10^−/−^ mice were immunized with ovalbumin (Ova) or heat shock protein 70 (HSP70), subsequently arthritis was induced and scored as described in figure 1. (A) Proteoglycan (PG)-specific IgG1 and IgG2a and (B) HSP70-specific total IgG production was measured by ELISA in sera obtained on day 52. Data in A and B are depicted relative to a standard of pooled sera from either arthritic mice or HSP70 immunized mice. (C) Antigen-specific proliferation of spleen cells harvested on day 52 was measured after 72 hours culturing in the presence of recombinant microbial HSP70, Ova or PG. Proliferation was measured by detection of ^3^H incorporation and depicted as stimulation index (CPM Antigen/CPM background). The mean background values (responses without antigen) were for the PBS control group 1705±339 and for the HSP70 group 1394±468. (D) After 72 hours incubation, antigen-induced IFN-γ excretion in the culture supernatants was analyzed by ELISA. Values represent the mean±SEM (n = 5 mice in each group). Open bars show the Ova immunized group and filled bars the HSP70 immunized group. Significant difference (p<0.05, Mann-Whitney U Test) is indicated by the asterisk.

HSP70 immunization increased the antigen-specific IL-10 production suggesting that IL-10 is important for the anti-inflammatory capacity of HSP70. To address whether protection in arthritis by HSP70 immunization was indeed IL-10 dependent, IL10^−/−^ mice received 100 µg HSP70 or Ova as control i.p. in DDA on day −10, followed by induction of arthritis by two injections with PG in DDA on day 0 and day 21. HSP70 immunization of IL10^−/−^ mice, in contrast to wild type mice (WT), did not reduce arthritis severity as compared to control, immunized IL10^−/−^ mice. In addition, there was no obvious difference in the mean maximum arthritis score or the day of onset of disease (Table 1). To study the effect of HSP70 immunization, in the absence of IL-10, on the B cell response, PG- and HSP70-specific antibody production was measured in the sera of IL10^−/−^ mice on day 53 after the first PG injection. Induction of arthritis resulted in a clear PG-specific antibody response as shown by elevated IgG1 and IgG2a levels in both treatment groups (Fig. 6A). However, in parallel with the arthritis scores, HSP70 immunization in IL-10^−/−^ mice did not influence this PG-specific response. As expected, HSP70 immunization enhanced the HSP70-specific IgG production (Fig. 6B). Next, induction and modulation of the antigen-specific T cell response by HSP70 treatment in IL-10^−/−^ mice were addressed. Therefore, spleen cells were harvested on day 53 followed by analysis of antigen-specific proliferation after *in vitro* restimulation, for 72 hours with PG, Ova or HSP70. ^3^H incorporation was depicted as stimulation index (CPM antigen/CPM background). The mean background values (responses without antigen) were for the PBS control group 1705±339 and for the HSP70 group 1394±468. Similar to the response in WT mice, immunization of IL10^−/−^ mice with HSP70 enhanced the proliferative response to HSP70 restimulation compared to control immunization with Ova. But, it did not have an effect on PG-specific proliferation (Fig. 6C). Furthermore, Ova specific proliferation was slightly increased in mice that received Ova. In addition, the effect of HSP70 immunization on antigen-specific IFN-γ secretion was studied, by ELISA in the culture supernatants. Also in IL10^−/−^ mice, immunization with HSP70 increased the production of IFN-γ after HSP70 restimulation. But, the enhanced PG-induced IFN-γ production, observed in WT mice, was not observed in IL10^−/−^ mice (Fig. 6D). Taken together, this indicated that HSP70 immunization of IL-10^−/−^ mice induced an HSP70-specific B and T cell response that, in the absence of IL-10, failed to suppress PGIA development and PG-specific pathogenic IgG2a production.

**Table 1:** Mean day of onset and maximum arthritis score in WT and IL10^−/−^ mice.

	WT		IL10^−/−^	
	Mean day of onset	Maximum arthritis score	Mean day of onset	Maximum arthritis score
control	31±2.0	4.6±1.6	33.6±1.3	5.4±1.1
HSP70	52±3.2 **	1.1±0.3 *	33.0±0.0	4.2±1.4

Arthritis was induced in WT BALB/c (n = 10 per group) or IL-10^−/−^ mice (n = 5 per group) by two immunizations with proteoglycan in the adjuvant DDA on days 0 and day 21. Ten days prior to the induction of arthritis mice were immunized with 100 µg HSP70 whereas control mice received 100 µg EGFP or Ova. Subsequently, development of arthritis symptoms was scored by clinical examination. Data are expressed as mean±SEM and are representative for at least two independent experiments. * p<0.05 and ** p<0.01 (HSP70 immunized WT mice compared to control WT mice by Mann Whitney U test).

## Discussion

The anti-inflammatory properties of HSP have been shown in several studies in both animal models and in patients suffering from inflammatory diseases [1], [5], [11], [12], [23], [24]. However, for further development of HSP for therapeutic application, it is essential to understand the mechanisms by which HSP affect chronic inflammatory disease in more detail. In this study we analyzed the protective potential of HSP70 in PGIA, a chronic model for RA, studied the T cell response induced by HSP70 immunization and addressed whether the presence of IL-10 was essential. Our data clearly show that pretreatment with HSP70 delayed arthritis onset and dramatically reduced disease severity both clinically and histologically. Furthermore, it increased the antigen-specific T cell response to both the protective HSP70 and the disease inducing PG. In IL-10 deficient mice HSP70 immunization did not suppress arthritis illustrating the IL-10 dependency of HSP70 induced immuno-regulation.

The immuno-regulatory effect of HSP70 immunization has been demonstrated earlier in arthritis models in rats [9], [25], [26]. In contrast to the rat models, the mouse PGIA model is a chronic and progressive model of RA [27]. In addition, in this model arthritis can be induced by immunization with proteoglycan mixed with synthetic adjuvant DDA instead of CFA [18]. Therefore, the possibility of interfering immune responses induced by mycobacterial HSP present in CFA can be excluded.

In the present study an HSP70-specific T cell response was induced upon HSP70 immunization as detected by increased proliferation, IL-10 and IFN-γ production upon HSP70 *in vitro* restimulation. Remarkably, also an enhanced PG-specific T cell response, with a similar cytokine profile, was found in HSP70 immunized mice. This might be explained by HSP70 interaction with innate receptors on antigen presenting cells, resulting in enhanced antigen priming to the subsequent PG immunization. Similarly, peptide-specific CD4^+^ T cell proliferation has been described to be enhanced when immunogenic peptides were fused to HSP70 subsequently resulting in increased immunogenicity of the peptides [28]. Alternatively, HSP70-induced regulatory T cells might induce a tolerogenic micro-milieu, by their cytokine production, enhancing the induction of regulatory T cells via infectious tolerance. This has been shown to occur in other models of autoimmunity in an IL-10 dependent manner [29].

Our data show that Hsp70 immunization induces a population of IL-10 producing T cells that do not express enhanced levels of Foxp3. Besides Foxp3 expressing CD25^+^ T cells, additional regulatory T cell subsets, such as adaptive Tr1, that do not express high levels of Foxp3 constitutively, are known [30]. Tr1 cells are induced in the periphery upon encounter of antigen and express high levels of IL-10 [31]. The HSP70-specifc IL-10 production together with enhanced IL-10 mRNA expression in CD4^+^CD25^+^ T cells in HSP70 immunized mice suggests activation or induction of HSP70-specific regulatory T cells. However, our observation that also CD3^−^ cells produce enhanced levels of IL-10 in HSP70 immunized mice (data not shown) indicates that multiple subsets of regulatory cells can contribute to regulation and this remains to be elucidated. This is in contrast to a recent study, where we showed an increase in Foxp3 expressing regulatory T cells, after HSP treatment, in a mouse atherosclerosis model [4]. However, in the present study CD4^+^CD25^+^ T cells from HSP70 immunized mice did not increasingly express Foxp3, indicating the possible presence of distinct types of regulation in different models of inflammation.

B cell responses play an important role in the pathogeneses of RA as demonstrated by the success of B cell targeted therapy, for example with monoclonal antibodies like Rituximab, as recently summarized [32]. Also in the PGIA model, B cells are important for disease induction [17], [33]. Therefore, the effect of HSP70 immunization on the B cell response was addressed by measuring PG-specific IgG1 and IgG2a production. Interestingly, decreased PG-specific IgG2a production was found in HSP70 immunized mice, while the IgG1 levels were not significantly changed. In earlier studies the IgG1 isotype has been shown to be the dominant isotype in the PGIA model. However, in agreement with our data, IgG2a levels have been described to correlate with disease severity [34]. Furthermore, the IgG2a isotype is known to be Th1 related. In accordance with these findings PGIA is considered to be a Th1 mediated model. Thus our data indicate that the pathogenic Th1 mediated PG-specific antibody response was inhibited by HSP70 immunization. In contrast to the arthritis associated PG-specific antibody response, HSP-specific antibodies have been shown to suppress adjuvant induced arthritis in rats [21], [22]. Therefore, the increased HSP70 IgG production found in our study in HSP70 immunized mice might be important for the regulatory potential of HSP70 treatment.

The increased HSP70-specifc B and T cell response, found in WT mice after HSP70 immunization, was also found in IL-10^−/−^ mice. However, in the absence of IL-10 this response failed to suppress the pathogenic PG-specific B cell response and it did not increase the PG-specific T cell response as observed in WT mice. So, despite the induction of an obvious HSP70-specific response, the protective effects of HSP70 immunization on the PG-specific B and T cell response were shown to be IL-10 dependent, consequently leading to development of severe arthritis in the absence of IL-10. The important role of IL-10 in arthritis has been studied previously in the PGIA model. In this study transfer of cells expressing IL-10 after retroviral IL-10 gene transduction could suppress arthritis [35]. Moreover, IL-10 has been described to stimulate PG synthesis and to reverse cartilage degradation induced by activated mononuclear cells [14] and it negatively correlated with progression of joint destruction in RA [36], showing the important anti-inflammatory function of IL-10 in human RA.

Due to their stress inducible nature HSP can be ideal candidates for immunotherapy against chronic inflammatory diseases like RA. HSP will be upregulated, locally at the site of inflammation in the joint, as reported previously [37]–[39]. Therefore, HSP-specific immune responses can be targeted specifically to sites of inflammation. Moreover, enhanced HSP-specific T cell responses in patients have been described [5], [6], [40]. The observation that such responses seemed to be associated with a benign form of disease, which over time preceded disease remission [5], [6], and the fact that HSP-mediated preventive and therapeutic immune interventions were effective in animal models of chronic inflammatory diseases, suggests the immunotherapeutic potential of HSP in patients with inflammatory autoimmune diseases.

In summary, this study shows that HSP70 can modulate inflammation in a model for chronic and progressive arthritis through IL-10 dependent mechanisms, operating via suppression of pathogenic antibody responses and the development of regulatory T cells. The chronic and progressive nature of the model and the possibility to use specific knock out mice, like the IL10^−/−^ used in this study, will enable further mechanistic studies needed for the development of HSP based immune intervention in chronic inflammatory diseases.
